# Monitoring Quality of Care in the WHO Africa Region-A study design for measurement and tracking, towards UHC attainment

**DOI:** 10.1080/16549716.2021.1939493

**Published:** 2021-07-28

**Authors:** Regina Titi-Ofei, Doris Osei-Afriyie, Humphrey Karamagi

**Affiliations:** a Data, Analytics and Knowledge Management Unit, WHO Regional Office for Africa, Brazzaville, Congo; b Department of Epidemiology and Public Health, Swiss Tropical and Public Health Institute, Basel, Switzerland; c University of Basel, Basel, Switzerland

**Keywords:** Health systems development, quality of care, universal health coverage, data analytics

## Abstract

This paper reports on the design of a study to generate a quality of care index for countries in the World Health Organization Africa Region.

Quality of care, for all people at all times, remains pivotal to the advancement of the 2030 agenda and the attainment of Universal Health Coverage. We present a study protocol for deriving a quality of care index, hinged on indicators and data elements currently monitored through routine information systems and institutionalized facility assessments in the World Health Organization Africa Region.

This paper seeks to offer more insight into options in the Region for strengthening monitoring processes of quality of care, as a step towards generating empirical evidence which can galvanize action towards an improved care process.

The methodology proposed in this study design has broad implications for policymaking and priority setting for countries, emphasizing the need for robust empirical measures to understand the functionality of health systems for the delivery of quality essential services. Application of this protocol will guide policymaking, as countries work to increasingly improve quality of care and adopt policies that will best facilitate their advancement towards Universal Health Coverage.

## Background

High quality of care for all people at all times remains pivotal to the advancement of the 2030 sustainable development goal (SDG) agenda and the attainment of Universal Health Coverage. In 2016, Across lower-middle income countries (LMICs), an estimated 8.6 million excess deaths were attributable to health care, of which five million were due to poor quality of care and the remaining due to non-utilization of health care [1]. In the World Health Organization (WHO) Africa Region, countries face challenges with poor quality of care which have been associated with the worsening outcomes of health, despite strong efforts and commitments to increase the scope of interventions available in the service delivery system, and access to them [2].

The elements of poor quality of care in the Region have been shown by many studies to be driven by issues such as poor staff attitudes towards clients, bureaucracy in facilities, infrastructural state of facilities, inadequacy of medical supplies and low patient satisfaction of services [3–8]. In addition, the content of the care during consultations by providers in the Region has been shown to be limited in scope [9].

Operating within these constraints, quality of care is further compromised by poor health sector governance, absence of communication networks within the process, hierarchical dynamics (which influence compliance to care protocols) and low levels of accountability system to users (poor operationalization of facility-based therapeutic committees and regulatory agencies to monitor the quality and safety of care) [10]. As a step to improving this, it is important that measurements for quality of care provided are embedded in health systems, with adequate improvements in their capacity to track these, for informed decision-making. However, measurements for quality of care for the entire Africa Region remain lacking [11], partly due to an absence of monitoring frameworks for standards of quality of care that can facilitate cross-country comparisons and are based on the regional context.

The aim of this paper is to offer additional insights into methodological approaches that can be leveraged by health decision makers in the Africa Region to generate measures of quality of care, towards improvements that are anchored on overall system functionality. The goal of this paper is therefore not to present a new conceptual framework for quality of care but rather to show the opportunity that exists to monitor quality of care in the context of the region’s health information systems.

### Defining and measuring quality of care

Quality of care is a multifaceted and complex intervention and to capture this complexity, *The Institute of Medicine* described quality of care as ‘the degree to which health services for individuals and populations increase the likelihood of desired outcomes and are consistent with current professional knowledge' [12].

To assess quality of care, various frameworks have evolved over time to emphasize the different components of quality of care. Our proposal for measuring quality of care in the Africa Region bases our analytical approach on the Donabedian model, a widely adopted model for quality of care [13]. The model conceptualizes quality of care along three main organizational dimensions: structure, process and outcomes that are connected by a unidirectional path, in that order. The structure dimension involves the attributes of the setting of care and inputs for service provision. This includes facility environment, equipment, staff training and provider’s knowledge. Process comprises of both the interactions between patients and providers and how care delivery is coordinated and performed. Finally, is the outcome dimension, which refers to the effects of health care on patients and populations. This involves the changes in health status, patient satisfaction and patient quality of life.

Although the Donabedian model has been widely used, it has been criticized for its failure to acknowledge critical characteristics such as governance and management, which are important enabling factors to consider for understanding quality of care. Other frameworks of quality of care have emerged recently to build on the Donabedian model, taking cognizance of the SDG agenda. *The Lancet Global Health Commission on High Quality Health Systems* proposes for the improvement of quality to be approached from a systems perspective, within a strong enabling environment, that allows for quality by leadership, across the various levels of the health system [14]. It proposes for the measurement of quality of care to focus on components related to the processes of care, which include competent care and systems as well as user experiences. Their framework provides a comprehensive approach to measure quality of care. However, health information systems in the Africa Region are not as robust to be able to comprehensively measure the various components proposed by the commission’s framework [15]. On the other hand, the Donabedian model offers policymakers the opportunity to draw on a widely accepted conceptual model that is appropriate for the current health information systems in the Region. It places an emphasis on the measurement of tangible elements of quality of care, which have a key value proposition for timely and immediate policy action, while health information systems continue to be strengthened and capacitated to expand measurement of a broader range of quality of care measures.

## Methodology

### Monitoring quality of care in the WHO Africa region: a proposal for a quality index

The WHO Africa Region has made a commitment to improving the quality of care of health systems in the Region as part of the framework of actions for progress in the attainment of UHC and health-related SDG targets [16,17]. The framework, which was endorsed by the Region’s Ministers of Health, at the *67^th^ Regional Committee Meeting*, provides three dimensions of quality of care to consider: *improving user experience in the care process, assuring patient safety, and improving the effectiveness of interventions,* which are described in Table 1. These aspects of the care process have become increasingly important in the context of the COVID-19 pandemic, where health facilities are faced with the need to adhere to strict infection prevention and control standards to ensure patient safety, efficient processes in patient triage, amongst other interventions needed to ensure patient safety and improvement of the care experience.

**Table 1.** Description of identified sub-domains for defining quality of care

Sub-domain	Description
User Experiences	The perceptions of how well the care process adhered to their expectations; this is what drives utilization, though not always correlated with the actual quality of care provided, given the subjectivity of this measurement, and its associated biases
Patient Safety	How well clients can avoid harm during the process of receiving care
Effectiveness of Interventions	How appropriate the interventions provided are for the care needs

### Selection of indicators for quality of care index

As a process to consolidate composite indicators for the proposed index, we subjected proposed indicators to expert consultations with Member States over a two-year (2016–18) process, involving the 47 countries of the region [16,18]. Additionally, a comprehensive search of the literature, and recognition of the limitations and capacities of the Region’s health information systems, was taken into consideration for the indicator selection process. In general, the indicators selected through the routine information systems are those that are widely available across all 47 Member States, to enable comparability of the index across the region, as well as those that are calculated on a routine basis to inform subnational and national level policy processes. In most countries, these are aligned with the WHO 100 core health indicators which provide key tracer indicators across various disease programs and the health system [19].

Within the health information system, data collected through facility-based health management information systems (HMIS), health facility assessments [20,21] and population surveys, and aligned with the three dimensions proposed in the regional framework of actions were considered. Health facility assessments are critical components of health system performance monitoring and a key source of information for national level policy and planning processes across the Africa Region [11,21,22]. They provide key information on general service readiness, availability and overall functionality of service provision units, within the health system, and are used widely to guide monitoring of health priorities and targets at national and subnational levels. In the context of COVID-19, the use of facility assessments has gained strong momentum as part of efforts to measure disruptions to essential health services increasingly taking place [23]. Within the ecosystem of institutionalized facility assessments, WHO supports the Service Availability and Readiness Assessments (SARA), The World Bank, the Service Delivery Indicators Survey (SDI), and USAID, the Service Provision Assessments Surveys (SPA). These have also been documented widely in peer-reviewed literature [11,24].

### Proposed matrix for monitoring quality of care

The focus of our proposed measurement lies at the input/structure, process and outcome dimensions. These are important, particularly in the African context, where improvement efforts have only begun, and therefore specific actionable guidance on where and how to invest for quality of care improvement are arguably more relevant than outcome level measures, which will become increasingly important as structural efforts to improve health systems quality across the region are strengthened [25].

We define measurement of quality of care across the Donabedian dimensions of quality, aligned with the sub-domains provided in the regional framework. For each sub-domain, a scan of the WHO Global Health Observatory, UN SDG database, national population-based surveys and the facility assessments was undertaken to identify relevant indicators that were fit for purpose and thematically aligned with each sub-domain. Based on the findings, we mapped the selected indicators along the various levels of this logic frame for each of the sub-domains of quality defined.

These identified indicators are proxies that conceptually represent the sub-capacities identified as critical for assessing quality of care. In total, nine indicators were identified: five for user experiences, two for patient safety and two for effectiveness of interventions. The final set of indicators generated are mapped out in Table 2.

**Table 2.** Proposed proxy indicators for a quality of care index in the WHO Africa Region

	User Experience	Patient Safety	Effectiveness of Interventions
INPUT	Basic Amenities Availability Score		
	Basic Equipment Availability Score		
	Essential Medicines Availability Score		
	Diagnostics Availability Score		
PROCESS	Satisfaction with Basic Health Services	Standard Precautions for Infection Prevention and Control	
OUTPUT		Still birth rate TB treatment success rate	Mortality from CVD cancer, diabetes or CRD between exact ages 30 and 70

### Protocol for data analysis

Recognizing that measurement of quality of care is driven by multiple factors that span various levels of the results chain (inputs/structure, processes and outcomes), we propose the derivation of a quality of care index that consolidates the information obtained from the various indicators, and provides a standardized summary measure of quality of care, comprehensible and useful for policy action. This index takes into account the fact that inputs alone are not a sufficient determinants of quality of care [26], and neither are outcome level measures.

A quality of care index fills a gap in the need for cross-country comparisons on status of quality of care and could galvanize action for quality improvement in the region’s health systems. We propose a systematically constructed index that can be subjected to statistical tests for validity, as well as sensitivity analyses to test its robustness [27].

### Data sourcing

The starting point is the assembly of the data. Data from various sources will be mapped out for the various indicators identified. Table 3 maps the standard data sources of the indicators identified for construction of the index.

**Table 3.** Standard data sources for indicators identified

Indicator	Source	Frequency	Level of collection
Basic Amenities Availability Score	Facility assessments	Periodic	Health Facility
Basic Equipment Availability Score	Facility assessments	Periodic	Health Facility
Essential Medicines Availability Score	Facility assessments	Periodic	Health Facility
Diagnostics Availability Score	Facility assessments	Periodic	Health Facility
General Service Readiness Score	Facility assessments	Periodic	Health Facility
Satisfaction with Basic Health Services	Facility assessments	Periodic	Health Facility
Standard Precautions for Infection Prevention and Control	Facility assessments	Periodic	Health Facility
Still birth rate	Population based surveys	Periodic	Population-based
TB treatment success rate	Population based surveys	Periodic	Population-based
Mortality from CVD cancer, diabetes or CRD between exact ages 30 and 70	Population based surveys	Periodic	Population-based

### Data normalization

Indicator values will be normalized from 0–100 (min-max) to make them suitable for aggregation. Normalization is achieved using the formula:

$$(X^{{\;}^{\prime }}=\frac{\left(x_{i}-x_{Minimum}\right)}{\left(x_{Maximum}-x_{Minimum}\right)})\times 100$$

Absolute goalpost values of 0–100 will be used in the normalization approach, thus, the values derived per unit will be relative to the identified minimum and maximum. This methodology is widely used in the construction of indices including the recent global health security index [28], the UHC service coverage index [29], the human development index [30], the SDG Index [31], among others.

### Deriving index values

Index values will be derived based on an arithmetic mean of all constituting indicators that will be calculated. Equal weights will be accorded to all component indicators, recognizing their equal importance for measuring quality of care in the context of the region’s health systems. Confidence intervals will be computed using calculated bias-corrected and accelerated percentile confidence intervals using 10,000 bootstrap replicates [32].

### Ascertaining construct validity

To ascertain convergent validity, index values will be correlated with outcome level measures of health, such as UHC service coverage. Beyond the outcome level, correlation with impact level measures of life expectancy, maternal mortality ratio and under-5 mortality rate can also be employed to test the face validity of the computed index. This is in line with approaches employed in other index computation studies, including Hogan et al., for the UHC Service Coverage Index and Karamagi et al., for assessing health systems functionality in the WHO Africa Region [2,29]. Furthermore, the validity of the weighting choice will be tested by calculating Spearman’s rank coefficients for the sub-indices defined – a low correlation coefficient validates the choice of equal weighting, thus ascertaining discriminant validity of the constituent sub-indices, which are proposed to measure different but complementary aspects of quality of care.

### Sensitivity analysis

A set of robustness tests, to assess the sensitivity of the computed index will be carried out. Proposed deterministic sensitivity tests including correlating an index constructed from an imputed data set vs a data set with missing values, correlation of index constructed by arithmetic mean vs geometric mean and finally, indicator drop analysis to assess the relative weights of the various constituent indicators to the overall quality of care index. The robustness of the index, irrespective of weighting scheme applied, can further be tested by the re-calculation of the index using differential weights generated through principal component analysis. This approach has been utilized widely in the computation of wealth indices in demographic health surveys [33,34]. A high correlation between both indices (equal vs differential weights), suggests that country ranking in the overall index is not sensitive to the choice of weights applied.

### Potential limitations

The proposed set of indicators for monitoring quality of care are presented, with the acknowledgement of its limitation in the inclusion of outcome level measures, including quality adjusted effective coverage measurements. Despite these being a best practice in the monitoring of quality of care in high-income settings, the development of an index based on input and process level indicators are a first step for monitoring quality, particularly in the context of the Africa Region, where widespread improvements in health information systems, and their capacity to monitor a wider range of indicators are still needed, without an additional burden of reporting [35]. We, however, propose some demand side measures, to emphasize the importance of a more robust set of measures for quality of care, beyond the traditional supply side indicators that have typically been found in the literature [36].

Furthermore, equity considerations are not applied in the selection of indicators and the proposed methodology for deriving the index. Subsequent efforts to consider all equity stratifiers in the development of a quality of care monitoring framework, ensuring that indicators selected are measureable at the subnational level, ensure that the data reveals disparities in the quality of care provided within and across countries.

Finally, our proposal for a quality index could infer the scoring of countries on a scale, a methodology which some oppose, given the varying contextual environments in which various service delivery systems operate. However, scoring has been shown to galvanize change, and serve as a call to action to poorer performing countries, to leverage the lessons learned from peers to improve [28].

### Options for scaling up measurements for quality of care

This proposal for a quality of care index for measuring health systems in the WHO Africa Region provides a starting point for how we leverage our health information systems, for tangible insights on how quality of care can be improved. We recognize there remains vast areas in need of improvement in the overall health information systems of countries. We recommend options in Table 4 on how data availability and quality can be improved for strengthened monitoring of the quality of care in the Africa Region.

**Table 4.** Options for improving data availability and quality for strengthened monitoring of the quality of care in the Africa Region

Invest in actionable National Quality of Care M&E Frameworks	
-	Galvanize political action and leadership to generate knowledge on the importance of increasing the scope of available measures for quality of care at country level
-	At national level, foster collaboration between health information directorates and quality of care directorates to scale up assessments of quality of care within the system
*Embed patient assessments on User Experiences in household population-based surveys*	
-	To strengthen the availability of data on process, incorporate into population-based surveys, to reduce the introduction of courtesy bias that is often associated with exit interviews for user experience assessments
*Scale up use of electronic health records which are vital opportunities for tracking quality of care process measures*	
-	Use of Electronic Health Records that can provide information on waiting times, and other patient-level indicators that can be used to assess quality
*Strengthen and routinize health facility assessment processes*	
-	Conduct facility assessments on a frequent basis to provide timely and relevant information for quality measurement. These should be deployed from a system perspective, vs the programme-specific facility surveys that currently fragments efforts

## Conclusion

Quality of care remains key for a well-functioning health system that can facilitate attainment of Universal Health Coverage. Quality of Care measurements remain sparse across the WHO African Region. This paper highlights an approach for assessing the quality of care provided by health systems across the WHO Africa Region, leveraging already existing indicators and data collection processes in countries. It presents an opportunity to identify and monitor important markers of the quality of care in the Africa Region, without placing a further burden of health data reporting on the region’s information systems [37].

The study design recognizes the need to advance these metrics to include more outcome level measures, as well as qualitative and intangible measures that can reveal additional aspects of the quality of care provided by systems. However, it provides a starting point, using a mix of indicators, where actionable insights can be identified by decision makers, ultimately towards improved population health outcomes.

The results derived from applying this protocol can provide preliminary direction on the concrete steps that need to be taken across countries for improved monitoring of quality of care towards UHC in the WHO Africa Region.

## Data Availability

Data for execution of study design are publicly available through WHO Global Health Observatory. https://www.who.int/data/gho

## Paper context

Quality of care (QoC) remains key for a well-functioning health system that can facilitate attainment of Universal Health Coverage. QoC measurements remain sparse across the WHO African Region. We propose a QoC index, hinged on data elements currently monitored though routine information systems and institutionalized facility assessments in the region. Application of such measures of health system functionality present an opportunity for monitoring health system performance to galvanize policy action towards improved health outcomes.
